# Advancing the diagnosis of pulmonary arterial hypertension-a Brazilian perspective aligned with global standards

**DOI:** 10.36416/1806-3756/e20250515

**Published:** 2026-03-05

**Authors:** Aaron B Waxman

**Affiliations:** 1. Pulmonary and Critical Care Medicine, Pulmonary Vascular Disease Program and Center for Pulmonary-Heart Diseases, Mass General Brigham - Brigham and Women’s Hospital, Harvard Medical School, Boston, MA, USA.

The Brazilian Thoracic Association’s comprehensive statement on pulmonary arterial hypertension (PAH) diagnosis, presented in this issue of the *Jornal Brasileiro de Pneumologia*, represents a significant step in aligning Brazilian clinical practice with the latest international evidence and guidelines.^1^ This thoroughly researched document is timely, integrating current hemodynamic definitions, diagnostic algorithms, and risk stratification frameworks while addressing region-specific considerations relevant to clinical care in Brazil.

A cornerstone of this statement is the adoption of the revised mean pulmonary artery pressure (mPAP) threshold of > 20 mmHg for defining pulmonary hypertension (PH).^1^ ^-^ ^3^ Introduced in the 2022 ESC/ERS guidelines and reinforced by the 2024 World Symposium on Pulmonary Hypertension, this paradigm shift is grounded in robust normative and prognostic data.^2^ ^-^ ^4^ Population studies have demonstrated that normal mPAP is around 14 ± 3 mmHg, and multiple 2023-2025 studies have confirmed that the new threshold captures patients with early disease who exhibit increased morbidity and mortality risk.^2^ ^,^ ^5^ ^,^ ^6^

The authors appropriately contextualize this within a diagnostic triad of pre-capillary PH: mPAP > 20 mmHg, pulmonary arterial wedge pressure (PAWP) ≤ 15 mmHg, and pulmonary vascular resistance (PVR) > 2 Wood Units.^2^ ^,^ ^3^ This integrated approach, supported by 2024 publications in high-impact journals, ensures that diagnosis reflects true pulmonary vascular pathology rather than isolated pressure elevations.^2^ ^,^ ^3^ ^,^ ^7^ Notably, patients with mPAP between 21-24 mmHg who were previously undiagnosed now benefit from earlier PH detection and monitoring, particularly in high-risk populations such as those with connective tissue disease.^8^ ^-^ ^10^

The statement’s detailed treatment of right heart catheterization (RHC) technique exemplifies best practices in invasive hemodynamic assessment.^1^ ^,^ ^11^ ^,^ ^12^ The authors correctly emphasize that RHC should be performed in expert centers where procedural standardization, accurate zero-reference positioning, and rigorous quality control minimize diagnostic error. Contemporary literature confirms that severe adverse event rates remain below 1.1% in experienced centers, underscoring the safety of this gold-standard diagnostic modality.^1^ ^,^ ^11^ ^,^ ^13^

The inclusion of guidance on fluid challenge, exercise hemodynamics, and vasoreactivity testing enhances the clinical utility of the document. Exercise RHC has gained renewed attention in recent years for detecting early disease and uncovering causes of unexplained dyspnea, with an mPAP/CO slope > 3 mmHg/L/min as the threshold for abnormal pulmonary vascular responses to exercise.^1^ ^,^ ^2^ ^,^ ^7^ Similarly, the detailed protocol for fluid challenge (500 mL saline at ~100 mL/min) to unmask occult left heart disease addresses a common diagnostic dilemma in clinical practice.

The statement’s comprehensive, multimodal approach to PAH diagnosis is particularly valuable. Integration of echocardiography, cardiac MRI, ventilation-perfusion scintigraphy, cardiopulmonary exercise testing, and laboratory biomarkers reflects the latest international recommendations and advances in diagnostic technology.^1^ ^,^ ^14^ ^,^ ^15^ Echocardiography is appropriately positioned as the most important noninvasive screening tool, with detailed discussion of tricuspid regurgitation jet velocity thresholds, right ventricular functional parameters (TAPSE, FAC, S’), and ventriculoarterial coupling (TAPSE/sPAP ratio).^2^ ^,^ ^16^ Recent studies validate the prognostic value of multiple integrated echocardiographic indices rather than isolated measurements.^1^ ^,^ ^14^ ^,^ ^15^

The document also acknowledges emerging technologies, including cardiac MRI for RV functional assessment and dual-energy CT as a potential alternative to V/Q scintigraphy for chronic thromboembolic PH (CTEPH) exclusion.^15^ A 2025 multimodal deep learning study demonstrated that integrating chest X-ray, ECG, echocardiography, and electronic health record data significantly outperforms single-modality approaches, achieving diagnostic ROC AUC as high as 0.96 in retrospective cohorts.^15^ While promising, these approaches remain exploratory, and not yet validated for routine clinical use. The statement’s forward-looking perspective on precision medicine and multimodal imaging positions Brazilian clinicians to adopt these innovations as evidence matures.^14^ ^,^ ^15^ ^,^ ^17^

The statement’s treatment of multiparametric risk stratification aligns with recent evidence from 2023-2025.^1^ ^,^ ^18^ The adoption of the ESC/ERS 3-strata model (low, intermediate, and high risk) with updated thresholds for 1-year mortality (low < 5%, intermediate 5-20%, and high > 20%) reflects contemporary registry data. The document appropriately includes hemodynamic parameters from diagnostic catheterization (right atrial pressure, cardiac index, stroke volume index, and mixed-venous oxygen saturation) alongside functional class, six-minute walk distance, and BNP/NT-proBNP levels, recognizing that no single variable suffices for prognostic information.^1^ ^,^ ^18^ Recent validation studies of risk calculators such as REVEAL 2.0 and four-strata models (subdividing intermediate risk into intermediate-low and intermediate-high) demonstrate improved sensitivity for detecting patients with worse prognosis during follow-up.^18^ ^-^ ^20^ The emphasis on longitudinal reassessment every 3-6 months and achieving low-risk status with treatment reflects the treat-to-target paradigm that is now a standard in PAH management.^18^ ^,^ ^19^

One of the most impactful contributions of this statement is the proposed framework for establishing and recognizing PH referral centers in Brazil. The five criteria-multidisciplinary expertise, diagnostic capabilities (with mandatory RHC), treatment resources, patient volume, and quality assurance-mirror international standards and address a critical gap in the Brazilian healthcare system.^21^ Recent studies from Latin America demonstrate substantial variability in RHC utilization, with expert centers achieving 92% RHC rates compared to 65% in non-specialized settings.^21^ This disparity underscores the urgent need for standardized center designation and referral pathways.^21^ ^,^ ^22^

Ensuring equitable access to appropriate diagnostic and therapeutic resources remains a critical challenge within the Brazilian healthcare system. Recent surveys reveal significant regional disparities in access to expert, specialized diagnostic testing (such as RHC and advanced imaging), and recommended PAH combination therapies.^21^ Geographic barriers, limited specialist availability, and formulary restrictions result in uneven care and suboptimal outcomes.^21^ ^,^ ^23^ The establishment of standardized referral networks and formal recognition of expert PH centers-alongside policies to expand drug availability-offers key solutions. Innovative models, including community-based health initiatives and telehealth programs, show promise in extending specialized care to remote and underserved areas, yet continued policy-level investment is required to realize universal access.^24^

Ultimately, advancing PAH diagnosis and management in Brazil depends not only on adopting best-evidence guidelines, but also on coordinated efforts to improve healthcare infrastructure, ensure timely referral to expert centers, and enhance therapeutic reach regardless of location or socioeconomic status.^21^ The statement’s call for comprehensive patient registries, outcome reporting, and clinical trial participation positions Brazilian centers to contribute meaningfully to the global evidence base while improving local care delivery.^14^ ^,^ ^17^

The statement acknowledges the promise of genetic testing, emerging biomarkers, and advanced imaging in enabling personalized PAH approaches. Advances in genomics (particularly *BMPR2* and *EIF2AK4* mutations), proteomics, multi-omics integration, and artificial intelligence are progressively transforming the diagnostic landscape.^14^ ^,^ ^15^ ^,^ ^17^ While genetic testing remains limited in Brazil’s public healthcare system, the statement prepares clinicians for future implementation and underscores the importance of family counseling and early detection in hereditary cases.^14^

This statement from the Brazilian Thoracic Association represents a rigorous, evidence-based, and highly practical contribution to PAH diagnosis and risk stratification. By integrating the latest hemodynamic definitions, emphasizing RHC procedural excellence, adopting multiparametric risk assessment, and proposing a framework for expert center designation, the authors have created an essential reference for Brazilian pulmonologists, cardiologists, and multidisciplinary teams. The document’s alignment with current international guidelines, incorporation of recent evidence, and attention to Brazilian-specific considerations (including schistosomiasis-associated PAH) demonstrate both scientific rigor and clinical relevance.^2^ ^,^ ^3^ ^,^ ^18^ ^,^ ^25^

As the PAH field evolves toward earlier diagnosis, precision phenotyping, and individualized treatment strategies, this statement positions Brazil to participate fully in these advances while ensuring that current best practices reach all patients. The emphasis on multidisciplinary collaboration, expert center development, and continued research will be critical to reducing diagnostic delays and transforming the prognosis of this challenging disease.^1^ ^,^ ^18^ ^,^ ^21^ ^,^ ^22^

## References

[1] Alves-Jr JL, Amado VM, Correa RA, Campos FAFT, Fernandes C, Ferreira EVM (2026). Diagnosis of pulmonary arterial hypertension a statement from the Brazilian Thoracic Association. J Bras Pneumol.

[2] Kovacs G, Bartolome S, Denton CP, Gatzoulis MA, Gu S, Khanna D (2024). Definition, classification and diagnosis of pulmonary hypertension. Eur Respir J.

[3] Kularatne M, Gerges C, Jevnikar M, Humbert M, Montani D (2024). Updated Clinical Classification and Hemodynamic Definitions of Pulmonary Hypertension and Its Clinical Implications. J Cardiovasc Dev Dis.

[4] Maron BA (2023). Revised Definition of Pulmonary Hypertension and Approach to Management A Clinical Primer. J Am Heart Assoc.

[5] Simonneau G, Hoeper MM (2019). The revised definition of pulmonary hypertension exploring the impact on patient management. Eur Heart J Suppl.

[6] Virsinskaite R, Karia N, Kotecha T, Schreiber BE, Coghlan JG, Knight DS (2023). Pulmonary hypertension - the latest updates for physicians. Clin Med (Lond).

[7] Sahay S, Lane J, Sharpe MG, Toth D, Paul D, Siuba MT (2023). Impact on Pulmonary Hypertension Hemodynamic Classification Based on the Methodology Used to Measure Pulmonary Artery Wedge Pressure and Cardiac Output. Ann Am Thorac Soc.

[8] Alturaif N, Alduraibi FK (2025). CTD-PAH an updated practical approach to screening, diagnosis, and management. Ther Adv Respir Dis.

[9] Dardi F, Bertozzi R, Ballerini A, Cennerazzo F, Donato F, Guarino D (2025). Phenotyping of patients with mild pulmonary hypertension. Open Heart.

[10] Zeder K, Brittain E, Kovacs G, Maron BA (2024). The Management of Mild Pulmonary Hypertension in Clinical Practice. Ann Am Thorac Soc.

[11] Guglielmi G, Krishnathasan K, Constantine A, Dimopoulos K (2024). Cardiac catheterization in pulmonary arterial hypertension Tips and tricks to enhance diagnosis and guide therapy. Int J Cardiol Congenit Heart Dis.

[12] Rosenkranz S, Preston IR (2015). Right heart catheterisation best practice and pitfalls in pulmonary hypertension. Eur Respir Rev.

[13] Manzi L, Sperandeo L, Forzano I, Castiello DS, Florimonte D, Paolillo R (2024). Contemporary Evidence and Practice on Right Heart Catheterization in Patients with Acute or Chronic Heart Failure. Diagnostics (Basel).

[14] Ricchi B, Jagana V, Singh E, Ochoa MT, Salamah J, Bisserier M (2025). Advances in diagnosis and patient profiling in pulmonary arterial hypertension for precision medicine. Ther Adv Respir Dis.

[15] Zhao W, Huang Z, Diao X, Yang Z, Zhao Z, Xia Y (2025). Development and validation of multimodal deep learning algorithms for detecting pulmonary hypertension. NPJ Digit Med.

[16] Mukherjee M, Rudski LG, Addetia K, Afilalo J, D'Alto M, Freed BH (2025). Guidelines for the Echocardiographic Assessment of the Right Heart in Adults and Special Considerations in Pulmonary Hypertension Recommendations from the American Society of Echocardiography. J Am Soc Echocardiog.

[17] Jain S, Kaur A, Qadeer A, Ghosh V, Thota S, Banala M (2025). Leveraging Artificial Intelligence for the Diagnosis of Systemic Sclerosis Associated Pulmonary Arterial Hypertension: Opportunities, Challenges, and Future Perspectives. Adv Respir Med.

[18] Dardi F, Boucly A, Benza R, Frantz R, Mercurio V, Olschewski H (2024). Risk stratification and treatment goals in pulmonary arterial hypertension. Eur Respir J.

[19] Cascino TM, McLaughlin VV (2021). Upfront Combination Therapy for Pulmonary Arterial Hypertension Time to Be More Ambitious than AMBITION. Am J Respir Crit Care Med.

[20] Lang IM, Gojic V (2024). Risk scoring in pulmonary hypertension One size fits all?. Eur J Heart Fail.

[21] Orozco-Levi M, Souza R, Bluro IM, Harley J, Hernández Oropeza JL, Lescano A (2024). Pathway to care, treatment and disease burden of pulmonary arterial hypertension a real-world survey of physicians and patients in Latin America. BMJ Open.

[22] Jansen SMA, Huis in't Veld AE, Tolen PHCG, Jacobs W, Willemsen HM, Grotjohan HP (2022). Clinical Characteristics of Patients Undergoing Right Heart Catheterizations in Community Hospitals. J Am Heart Assoc.

[23] Fernandes CJCDS, Salibe-Filho W, Alves-Jr JL, Jardim CVP, Vieira TM, Terra-Filho M (2025). Riociguat for CTEPH A retrospective cohort study from a single reference center in Brazil. Clinics (Sao Paulo).

[24] Lamas CA, Santana Alves PG, Nader de Araújo L, de Souza Paes AB, Cielo AC, Maciel de Almeida Lopes L (2025). Telehealth Initiative to Enhance Primary Care Access in Brazil (UBS+Digital Project) Multicenter Prospective Study. J Med Internet Res.

[25] Humbert M, Kovacs G, Hoeper MM, Badagliacca R, Berger RMF, Brida M (2023). 2022 ESC/ERS Guidelines for the diagnosis and treatment of pulmonary hypertension. Eur Respir J.

